# Loss of Numb promotes hepatic progenitor expansion and intrahepatic cholangiocarcinoma by enhancing Notch signaling

**DOI:** 10.1038/s41419-021-04263-w

**Published:** 2021-10-19

**Authors:** Yuke Shu, Qing Xu, Yahong Xu, Qing Tao, Mingyang Shao, Xiaoyue Cao, Yuwei Chen, Zhenru Wu, Menglin Chen, Yongjie Zhou, Ping Zhou, Yujun Shi, Hong Bu

**Affiliations:** 1 Institute of Clinical Pathology, Key Laboratory of Transplant Engineering and Immunology, NHC, West China Hospital, Sichuan University, Chengdu, 610041 China; 2 Laboratory of Transplantation, West China Hospital, Sichuan University, Chengdu, 610041 China; 3 Department of Pathology, Sichuan Tumor Hospital, Chengdu, 610041 China; 4 Department of Pathology, West China Hospital, Sichuan University, Chengdu, 610041 China

**Keywords:** Cancer, Liver cancer

## Abstract

Numb, a stem cell fate determinant, acts as a tumor suppressor and is closely related to a wide variety of malignancies. Intrahepatic cholangiocarcinoma (iCCA) originates from hepatic progenitors (HPCs); however, the role of Numb in HPC malignant transformation and iCCA development is still unclear. A retrospective cohort study indicated that Numb was frequently decreased in tumor tissues and suggests poor prognosis in iCCA patients. Consistently, in a chemically induced iCCA mouse model, Numb was downregulated in tumor cells compared to normal cholangiocytes. In diet-induced chronic liver injury mouse models, Numb ablation significantly promoted histological impairment, HPC expansion, and tumorigenesis. Similarly, Numb silencing in cultured iCCA cells enhanced cell spheroid growth, invasion, metastasis, and the expression of stem cell markers. Mechanistically, Numb was found to bind to the Notch intracellular domain (NICD), and Numb ablation promoted Notch signaling; this effect was reversed when Notch signaling was blocked by γ-secretase inhibitor treatment. Our results suggested that loss of Numb plays an important role in promoting HPC expansion, HPC malignant transformation, and, ultimately, iCCA development in chronically injured livers. Therapies targeting suppressed Numb are promising for the treatment of iCCA.

## Introduction

Primary liver cancer (PLC) is the sixth most commonly diagnosed cancer and the third leading cause of cancer death worldwide according to the latest global cancer statistics [1]. Intrahepatic cholangiocarcinoma (iCCA) accounts for 10–15% of PLCs, ranking as the second incidence rate of PLC after hepatocellular carcinoma (HCC). The incidence of iCCA has gradually increased in recent years [2–4]. Due to the occult incidence, early metastasis, and low resection rate, only 10%–15% of iCCA patients can receive radical resection [5–7]. Thus, it is urgent to dissect the precise molecular mechanisms of iCCA pathogenesis for targeted prevention and treatment.

Although the pathogenesis of iCCA is still not completely clear, iCCA often shares many risk factors with HCC, such as liver cirrhosis and viral hepatitis [3, 8]. In addition, cholestasis, parasite infection, radiation exposure, microenvironment damage, and metabolic syndrome are also closely correlated with the development of iCCA [7, 9, 10].

Almost all human liver diseases are accompanied by a certain degree of chronic liver injury. Hepatic progenitor cells (HPCs), the stem cells in the liver, expand rapidly upon diverse chronic injuries, leading to a characteristic histopathological change known as a ductular reaction [11]. HPCs possess self-renewal, bipotency, and strong proliferation ability, and they highly express embryonic liver markers, such as cytokeratin 19 (CK19), Sox9, EpCAM, CD133, CD24, and CD44 [12, 13]. HPC expansion is often associated with liver fibrosis because activated HPCs secrete growth factors, such as PDGF, TGF-β, and VEGF, leading to the activation of hepatic stellate cells (HSCs) and the deposition of extracellular matrix [14]. HPCs also recruit Kupffer cells, and in turn, Kupffer cells, together with activated HSCs, further promote HPC expansion [14]. HPCs have the bipotency to differentiate into hepatocytes or biliary epithelial cells [3, 15], and they have been demonstrated to give rise to both HCC and iCCA [16, 17].

Numb, a membrane protein, was first identified in the cleavage of Drosophila neurons. Acting as an important determinant of stem cell fate, Numb regulates the asymmetric mitosis of cells [18]. Numb is a negative regulator of the Notch signaling pathway, which controls the differentiation of stem cells, including HPCs. Activated Notch signaling promotes HPCs to differentiate into bile duct cells; in contrast, HPCs differentiate into hepatocytes when Notch signaling is blocked [19]. Numb is also related to a variety of malignant tumors. Generally, as a tumor suppressor gene, Numb is downregulated in a wide variety of malignancies, including non-small cell lung cancer, breast cancer, esophageal squamous cell carcinoma, and prostate cancer [20–22].

However, the expression and function of Numb in iCCA have not been explored. In the present study, we explored the role and underlying mechanisms of Numb in HPC proliferation, HPC malignant transformation, and, ultimately, the development of iCCA.

## Methods and materials

### Patient tissues

For the retrospective cohort study, human iCCA tissues resected from 121 diagnosed patients with complete clinical information were obtained from the West China Hospital of Sichuan University between 2009 and 2015. According to the signal distribution and intensity, the expression level of Numb was scored by three pathologists using a blinded method. Briefly, at least five 400× magnified areas were examined and scored for signal distribution as follows: 0, <5% stained; 1, 5–25% stained; 2, 25–75% stained; and 3, >75% stained. The intensity of staining was scored as follows: 1, weak; 2, moderate; and 3, intense. Tissues with an IHC score (product of signal distribution score and staining intensity score) of 0–3 were designated tissues with low expression, and those with scores of 4–9 were designated tissues with high expression. Another eight iCCA samples with paired paracancerous tissues used for western blotting analysis were rapidly frozen and stored in liquid nitrogen after removal until protein extraction. All patient materials were obtained with written informed consent. Approval for this study was granted by the Ethics Committee of the West China Hospital, Sichuan University. The antibodies used in this study are listed in Supplementary Table 1.

### Animals and treatments

Numb^loxP/loxP^ and Alb-cre mice were generated by crossing mice harboring a LoxP-flanked allele of Numb (Numb^loxP/loxP^ mice) on a C57BL/6 J background (Jackson Laboratory) with Alb-cre mice on a C57BL/6 J background (Shanghai Biomodel Organism Science & Technology Development Co., Ltd., China). In Numb^loxP/loxP^ and Alb-cre mice (hereinafter referred to as Numb^−/−^ mice), the Numb gene in the progeny cells of hepatoblasts, including hepatocytes and cholangiocytes, begins to be ablated at E8.5 and is completely deleted ~1 month after birth. Littermate Numb^loxP/loxP^ mice without Cre were used as wild-type controls (WT).

To establish an HPC expansion model, six-week-old male mice were fed a DDC (0.1% 3,5-diethoxycarbonyl-1,4-dihydrocollidine) diet for 4 weeks to induce HPC production [23]. To establish an iCCA model, six-week-old male mice were treated with thioacetamide (TAA, 300 mg/L in drinking water), a confirmed carcinogen, for 4–6 months to induce tumorigenesis in Numb^−/−^ mice or WT mice [24]. Over ten mice per group were sacrificed at the indicated time points. All mice were housed with corncob bedding under SPF (specific pathogen-free) conditions. The animal care and experimental procedures were conducted in accordance with national and international laws and policies and were approved by the Animal Care and Use Committee of Sichuan University. The sequences of genotyping primers are presented in Supplementary Table 2.

### Statistical analysis

Statistical analyses were performed using Microsoft Excel software or Prism GraphPad 8. The results were expressed as the means ± SDs. Kaplan–Meier analysis was used to determine the recurrence-free survival and overall survival of patients, and the log-rank test was used to investigate differences. Differences between unpaired groups with normal distributions and variance homogeneity were compared using Student’s *t*-test, and differences between unpaired groups with normal distributions but without variance homogeneity were compared using Welch’s *t*-test. Differences between unpaired groups with nonnormal distributions were compared using Student’s *t*-test or the Mann–Whitney *U*-test. The significance of the differences between groups was tested using an unpaired, two-tailed Student’s *t*-test with Welch correction. A *P* value <0.05 was considered significant.

For more details, see the Supplementary Material.

## Results

### Numb is downregulated in iCCA and indicates poor prognosis

**Fig. 1:**
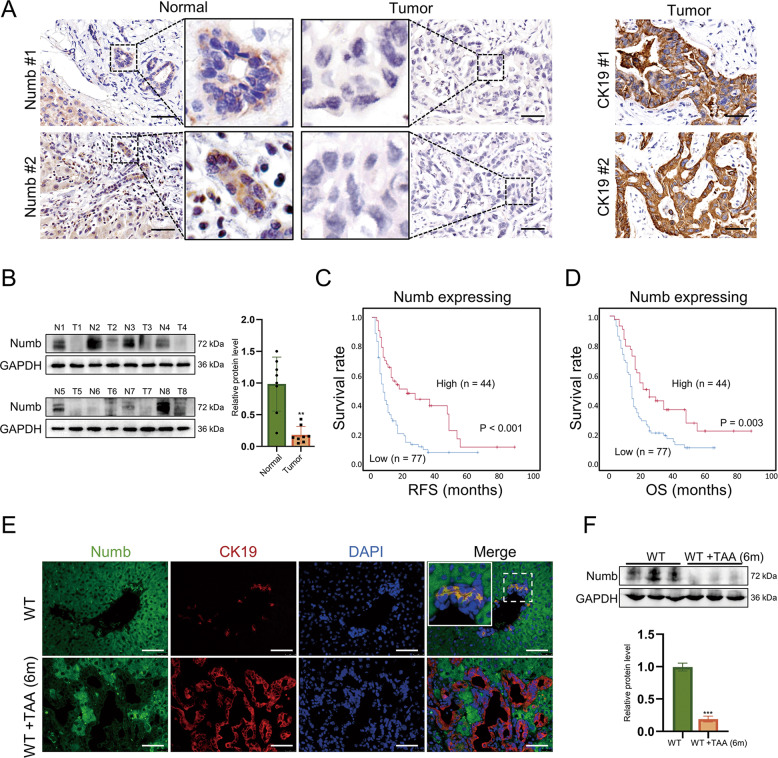
Numb is downregulated in human iCCA and mouse iCCA. **A** Immunohistochemistry staining of Numb expression in iCCA specimens and paired normal tissues. CK19 staining confirmed the cholangiocyte origins of the tumors. Scale bar, 50 μm. **B** Western blotting showing the different Numb protein levels between two pairs of iCCA tissues and adjacent nonmalignant tissues. The corresponding statistical analysis is also presented. **C**, **D** Correlation of Numb expression with the recurrence-free survival (RFS) time and the overall survival (OS) time of iCCA patients, respectively. **E** Immunofluorescence staining and **F** Western blotting showing that Numb was downregulated in mouse iCCA tissues (TAA treated for 6 months) with the corresponding statistical analysis. Scale bars, 50 μm. Data represent the mean ± SD of at least three independent experiments; *P < 0.05, **P < 0.01, and ***P < 0.001.

We assessed Numb expression in iCCA tissues and paired adjacent noncancerous tissues by IHC and immunofluorescence staining. In the noncancerous tissues, Numb was positively expressed in both hepatocytes and cholangiocytes; however, in the iCCA tissues, Numb was markedly downregulated as indicated by positive CK19 staining but negative Numb staining (Fig. 1A and Fig. S1A). Consistently, western blotting analysis demonstrated an extensive reduction in Numb in iCCA tissues (Fig. 1B). Although Numb was strongly downregulated in iCCA, the iCCA patients were into the following two groups according to IHC staining: high Numb expression group (Numb^High^, score 4–9) and low Numb expression group (Numb^Low^, score 1–3) (Fig. S1B). The correlation analysis between Numb expression and clinicopathological features of iCCA patients is shown in Supplementary Table 3. The median age of the patients was 56.36 ± 10.28 years in the Numb^Low^ group and 56.93 ± 10.23 years in the Numb^High^ group. There were mainly females (54.79%) in the Numb^Low^ group, but there were mainly males (63.64%) in the Numb^High^ group. The tumor size was 6.20 ± 2.75 cm in the Numb^Low^ group and 6.41 ± 2.43 cm in the Numb^High^ group. Patients with multiple tumors accounted for 10.95% in the Numb^Low^ group and 18.18% in the Numb^High^ group. There was a significant negative correlation between Numb expression and tumor differentiation. Importantly, Numb^Low^ iCCA patients displayed significantly shorter recurrence-free survival (RFS) and overall survival (OS) than Numb^High^ patients (Fig. 1C, D).

### Numb is downregulated in mouse iCCA

To explore the role of Numb in iCCA development, we first constructed a mouse iCCA model by feeding male mice aged 6 weeks with a TAA diet (300 mg/L in drinking water for 6 months). TAA is a confirmed carcinogen, and chronic TAA treatment in mice leads to prominent DR, periductular fibrosis, and, ultimately, iCCA [24]. Consistent with the reduction in Numb in human iCCA tissues, immunofluorescence staining showed that Numb was significantly inhibited in chemically induced mouse iCCA compared to noncancerous tissues, which was further confirmed by western blotting analysis (Fig. 1E, F).

### Loss of Numb promotes HPC expansion and liver fibrosis

As iCCA has long been suggested to be derived from HPCs [16, 17], we explored the role of Numb in HPC activation. We constructed a Numb knockout (Numb^loxP/loxP^ and Alb-cre, further abbreviated as Numb^−/−^) mouse model, and littermates without Cre were used as WT controls [25, 26]. Genotyping and western blotting analysis showed that Numb^−/−^ mice were successfully constructed, and the protein expression level of Numb was downregulated by more than 90% (Fig. S2A, B). Numb was ablated in both hepatocytes and cholangiocytes in the adult liver. To our surprise, Numb^−/−^ mice were born normally, and no defects or abnormalities were observed in liver development, histology, or metabolism (Fig. S2C–I), indicating that Numb ablation has no obvious effect on liver development and homeostasis. Most strikingly, Numb ablation did not impair liver regeneration following 70% partial hepatectomy (Fig. S3).

**Fig. 2:**
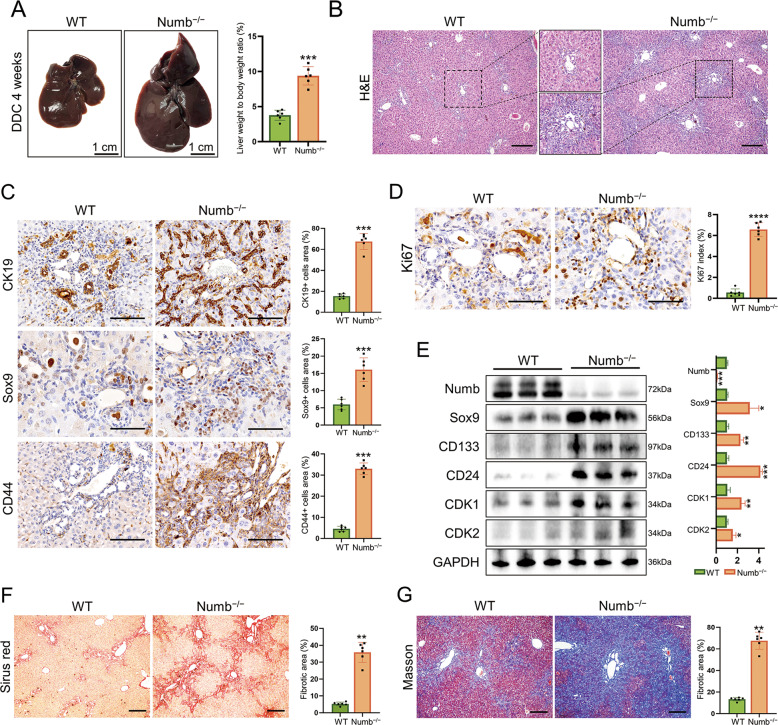
Numb ablation promotes HPC expansion and liver fibrosis. **A** Gross views and the liver weight/body weight ratios of WT and Numb^−/−^ livers after 4 weeks of DDC exposure. **B** Histological alterations were assessed with H&E staining. Scale bar, 200 μm. **C** Expression of HPC markers (CK19, Sox9, and CD44) and corresponding statistical results. Scale bars, 100 μm (CK19 and CD44) and 50 μm (Sox9). **D** Ki67 immunohistochemistry staining showing the active proliferation of HPCs in Numb^−/−^ livers. Scale bar, 50 μm. **E** Western blotting showing elevated progenitor markers (CD133, Sox9, and CD24) and critical cell cycle proteins (CDK1 and CDK2), and the relative expression of these proteins was also semiquantified and analyzed. **F**, **G** Masson trichrome and Sirius red staining were performed to measure liver fibrosis. Scale bar, 200 μm. All data represent the mean ± SD of at least three independent experiments; *P < 0.05, **P < 0.01, and ***P < 0.001.

To establish an HPC expansion model, we fed male mice aged 6 weeks with a 0.1% DDC-supplemented diet for 4 weeks [23]. Gross examination of the livers of DDC-treated mice revealed typical characteristics, including large size and dark brown color. In contrast, the livers of Numb^−/−^ mice were much larger in size and deeper brown in color, and Numb^−/−^ mice exhibited a higher liver to body weight ratio (Fig. 2A). Histologically, the significant ductular reaction was found in the portal area in Numb^−/−^ livers compared to their WT counterparts, indicating more intensive expansion of HPCs in the Numb^−/−^ livers (Fig. 2B). These findings were further confirmed by the increased CK19-, Sox9-, and CD44-labeled HPCs in Numb^−/−^ mice (Fig. 2C, S4). In addition, HPCs possessed strongly elevated proliferation ability based on Ki67 staining when Numb was knocked out (Fig. 2D). Western blotting also demonstrated the elevated expression of progenitor markers and cell cycle markers in Numb^−/−^ mice (Fig. 2E). In addition, Masson’s trichrome and Sirius red staining demonstrated more serious liver fibrosis in Numb^−/−^ mice than in WT mice (Fig. 2F, G). Collectively, these findings demonstrated that Numb plays a critical role in suppressing HPC expansion and periportal fibrosis upon chronic biliary injury.

### Numb ablation promotes the development of iCCA

**Fig. 3:**
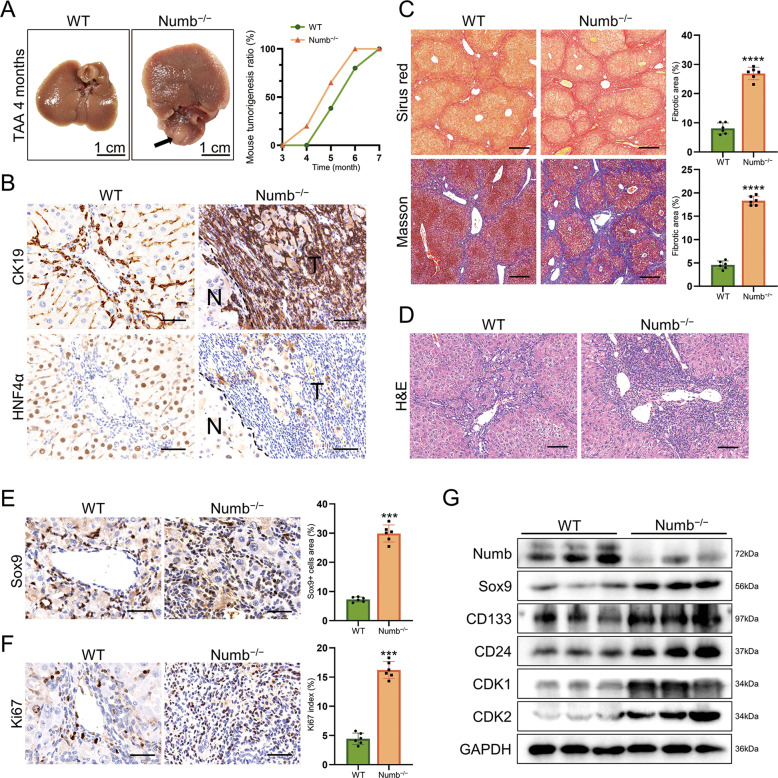
Numb ablation promotes HPC expansion, periportal fibrosis, and iCCA. **A** Gross views of WT and Numb^−/−^ livers after 4 months of TAA exposure and the tumorigenesis ratio of WT and Numb^−/−^ mice at specific times. The black arrowhead indicates the tumor nodule in the Numb^−/−^ liver. **B** CK19 and HNF4α immunohistochemistry staining showing that the tumor was cholangiocarcinoma. Scale bars, 50 μm. **C** Masson trichrome and Sirius red staining were performed to measure liver fibrosis. Scale bar, 200 μm. **D** Histological alterations were assessed with H&E staining. Scale bar, 200 μm. **E**, **F** Immunohistochemistry staining of Sox9 and Ki67 in WT and Numb^−/−^ livers, respectively. Scale bars, 25 μm. The corresponding statistical results are also shown. **G** Western blotting showing the elevated expression of progenitor markers (CD133, Sox9, and CD24) and critical cell cycle-related proteins (CDK1 and CDK2), and the relative expression of these proteins was also semiquantified and analyzed. All data represent the mean ± SD of at least three independent experiments; *P < 0.05, **P < 0.01, and ***P < 0.001.

Numb has long been regarded as a tumor suppressor. To further evaluate the role of Numb in iCCA development, we treated Numb^−/−^ mice with a TAA diet. After 4 months of TAA administration, tumor nodules developed in Numb^−/−^ livers, and the tumors in Numb^−/−^ livers occurred 2 months faster than that in WT livers with TAA administration (Fig. 3A). The tumor cells strongly expressed CK19 (bile duct epithelial cell marker) but not HNF4α (hepatocyte markers), and they lacked tubular structures, indicating that the tumor was a poorly differentiated iCCA (Fig. 3B). Similar to the histological findings observed in the DDC models, loss of Numb aggravated the liver injury and fibrosis in the nontumorous tissues caused by TAA administration (Fig. 3C, D). A high frequency of Sox9- and Ki67-positive iCCA cells demonstrated the high stemness and proliferation of this malignancy (Fig. 3E, F). Consistently, in the nontumorous tissues in the Numb^−/−^ livers, progenitor markers (Sox9, CD133, and CD24) and cell cycle proteins (CDK1 and CDK2) were significantly upregulated (Fig. 3G), further demonstrating that Numb deficiency promotes the expansion and malignant transformation of HPCs upon carcinogen challenge, which ultimately leads to the occurrence of iCCA.

### Numb knockdown promotes proliferation, metastasis, and stemness in iCCA cells

**Fig. 4:**
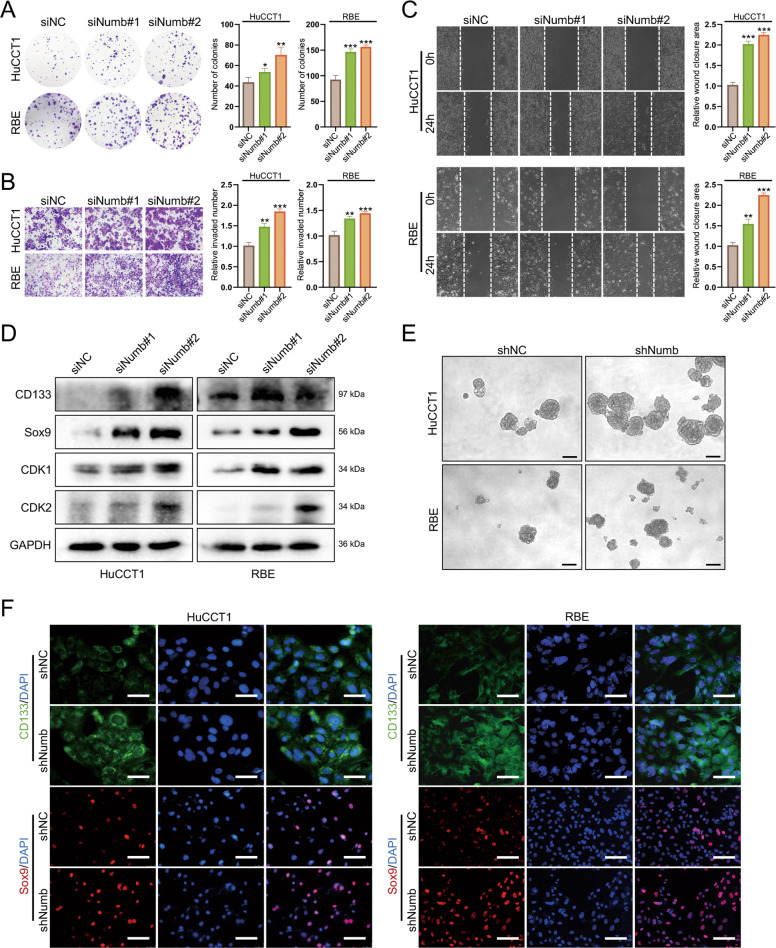
Numb knockdown promotes proliferation, metastasis, and stemness in iCCA cells. The colony formation assay (**A**), migration assay (**B**), and wound-healing assay (**C**) were performed in HuCCT1 and RBE cell lines. **D** Progenitor markers (CD133, Sox9, and CD24) and critical cell cycle proteins (CDK1 and CDK2) were upregulated after Numb silencing in HuCCT1 and RBE cells. **E** Sphere formation assays comparing the stemness of HuCCT1 and RBE cells after Numb silencing. Scale bar, 100 μm. **F** Immunofluorescence staining showing the upregulation of progenitor markers CD133 and Sox9 after Numb silencing. Scale bars, 50 μm (CD133) and 100 μm (Sox9). All data represent the mean ± SD of at least three independent experiments; *P < 0.05, **P < 0.01, and ***P < 0.001.

To further investigate the role of Numb in HPC expansion and iCCA development, we used siRNA to silence the Numb gene in the HuCCT1 and RBE human iCCA cell lines, which share many features with HPCs, including spheroid growth and expression of the stem cell markers, CK19 and CD133 [27]. The efficiencies of Numb attenuation were confirmed by western blotting (Fig. S5A). Based on colony formation assays, Transwell assays, and wound-healing assays, Numb knockdown significantly promoted iCCA cell proliferation, invasion, and migration in HuCCT1 and RBE cells (Fig. 4A–C). Western blotting results showed that the progenitor markers, CD133 and Sox9, and the cell cycle markers, CDK1 and CDK2, were significantly upregulated after Numb silencing (Fig. 4D).

To further investigate the effect of Numb on the stemness of iCCA cells, shRNA was transfected into a lentiviral vector (shNumb) to stably suppress Numb expression (Fig. S5B). These two cell lines were also transfected with an empty vector as a negative control (shNC). HuCCT1 and RBE cells formed an increasing number of spheres with stem cell characteristics after Numb silencing (Fig. 4E). In addition, immunofluorescence staining showed that the stem cell markers, CD133 and Sox9, were significantly upregulated after Numb silencing (Fig. 4F). Collectively, these findings indicated that Numb ablation promotes the growth, metastasis, and stemness of iCCA cells in vitro.

### Numb deficiency promotion of iCCA is dependent on Notch signaling

We next determined the role and signaling pathway by which Numb deficiency promotes iCCA development. To investigate how the knockdown of Numb promotes malignant transformation of ductular cells, we performed RNA-seq on nontumorous liver tissues from 4-month-old TAA-treated Numb^−/−^ mice and WT mice.

**Fig. 5:**
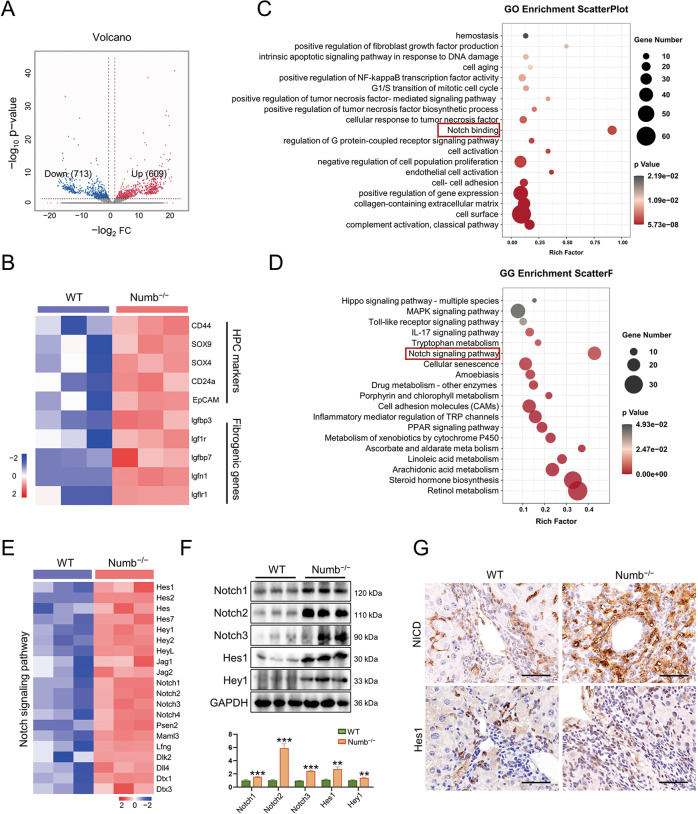
Numb deficiency promotion of iCCA is dependent on Notch signaling. **A** Volcano plot displaying the differentially expressed genes between WT and Numb^−/−^ mouse nontumorous liver tissues. **B** Expression of HPC markers and profibrogenic genes. **C**, **D** GO analysis and KEGG analysis showing that the differentially expressed genes were enriched in the Notch signaling pathway. **E** Alterations in the Notch signaling pathways between WT livers and Numb^−/−^ livers after TAA treatment. **F** Western blotting analysis of Notch-associated proteins and the key downstream proteins of Notch, NICD, and Hes1. **G** NICD and Hes1 immunohistochemistry staining in WT and Numb^−/−^ mouse livers. Scale bar, 50 μm. All data represent the mean ± SD of at least three independent experiments; *P < 0.05, **P < 0.01, and ***P < 0.001.

The gene expression profiles of livers from 4-month-old TAA-treated mice showed 713 downregulated genes and 609 upregulated genes in Numb^−/−^ mice compared to WT mice (Fig. 5A). Among these genes, progenitor marker genes, such as EpCAM, Sox4, Sox9, Cd44, and Cd24a, as well as profibrogenic genes, such as Igfbp7, Igfbp3, Igf1r, Igfn1, and Igflr1 [28], were significantly upregulated in nontumorous tissues in the Numb^−/−^ liver (Fig. 5B). Gene Ontology (GO) term analysis and KEGG analysis of the differentially regulated genes showed that the Notch signaling pathway was altered (Fig. 5C, D). Up to 20 genes related to Notch signaling, such as Hes1, Hey1, Heyl, Notch1, Notch2, Notch3, and Notch4, were upregulated in Numb^−/−^ mice. Correspondingly, western blotting identified that the expression of Notch-associated proteins, especially Notch2, was upregulated (Fig. 5E). Histologically, we found that the key downstream proteins of Notch, such as the Notch intracellular domain (NICD) and Hes1, were upregulated in Numb^−/−^ mice. Notably, NICD, which generally functions in nuclear translocation [29], was significantly upregulated in Numb^−/−^ livers, particularly in the nucleus (Fig. 5F). These findings suggested that Numb inactivation might promote iCCA via a canonical Notch signaling pathway.

### Numb regulates Notch signaling by binding to NICD

**Fig. 6:**
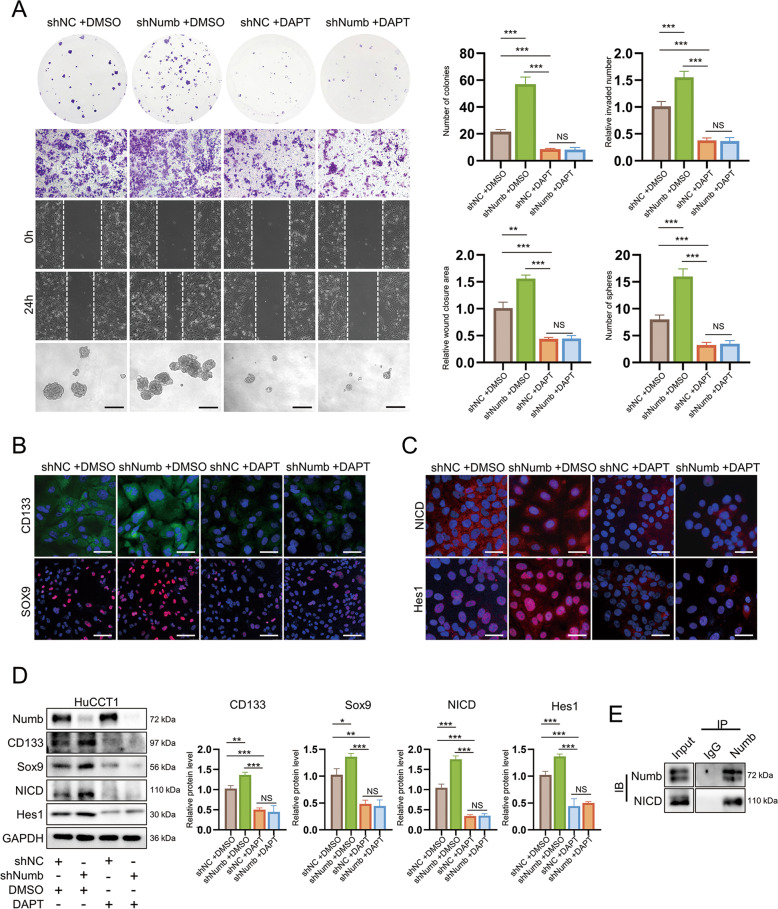
Numb regulates Notch signaling by binding to NICD in vitro. **A** Proliferation, metastasis, and stemness were measured by colony formation, migration, wound-healing, and sphere formation assays after DAPT treatment at a dose of 150 μM (the control group was treated with the same volume of DMSO without DAPT) in HuCCT1 cells. Scale bar, 200 μm. **B** Immunofluorescence staining of HPC markers (CD133 and Sox9) in HuCCT1 cells. Scale bars, 50 μm (CD133) and 100 μm (Sox9). **C** Immunofluorescence staining of the key proteins of Notch signaling, NICD, and Hes1, in HuCCT1 cells. Scale bar, 50 μm. **D** Western blotting displaying the upregulation of HPC markers and the downstream factors of Notch after Numb silencing was reversed after DAPT treatment in HuCCT1 cells. **E** Coimmunoprecipitation (CoIP) showing the direct binding of Numb and NICD. All data represent the mean ± SD of at least three independent experiments; *P < 0.05, **P < 0.01, and ***P < 0.001.

To further confirm the relationship between Numb expression and Notch signaling, DAPT, a specific γ-secretase inhibitor that mimics canonical Notch loss-of-function mutations by chemically preventing NICD cleavage [30], was used to treat HuCCT1 and RBE cells (150 μM). Numb knockdown promoted colony formation, metastasis, and sphere formation in HuCCT1 and RBE cells, whereas these changes were reversed by DAPT treatment (Figs. 6A and S6A). In addition, the upregulation of the stem cell markers, CD133 and Sox9, caused by Numb silencing was also reversed by DAPT (Figs. 6B and S6B). NICD was mostly located in the cytoplasm in HuCCT1 and RBE cells (Figs. 6C and S6C), and Numb knockdown resulted in high expression and nuclear accumulation of NICD; however, DAPT treatment eliminated the difference in NICD expression and location between the shNumb group and the corresponding NC group (Figs. 6C and S6C). Another key protein of Notch signaling, Hes1, was upregulated after silencing Numb; similarly, DAPT treatment returned Hes1 expression to a low level with no significant difference between the shNumb group and the NC group (Figs. 6C and S6C). Similar results were found by western blotting (Figs. 6D and S6D). Consistent with emerging findings, our results indicated that the role of Numb in promoting iCCA is largely dependent on Notch signaling.

**Fig. 7:**
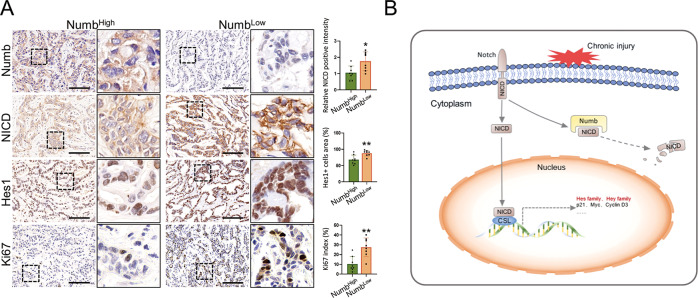
Negative correlation between Numb and NICD expression in human iCCA. **A** Immunohistochemistry staining of Numb, NICD, Hes1, and Ki67 in Numb^High^ iCCA samples and Numb^Low^ iCCA samples. Scale bar, 100 μm. **B** Diagram of the hypothetical roles of Numb in HPC expansion, liver fibrosis, and iCCA development by regulating the Notch signaling pathway. All data represent the mean ± SD of at least three independent experiments; *P < 0.05, **P < 0.01, and ***P < 0.001.

The high expression and nuclear accumulation of NICD after Numb silencing in both mice and iCCA cell lines (Figs. 5H, 7C, and S6C), together with the GO term analysis result displaying consistent upregulation of Notch-binding genes (Fig. 5D), prompted us to investigate whether there is an interaction between NICD and Numb in iCCA cells. Coimmunoprecipitation (CoIP) experiments using RBE cells showed that Numb is directly bound to NICD (Fig. 6E), which suggested that Numb may be involved in mediating the activity or nuclear translocation of NICD, thus regulating Notch signaling in iCCA. This hypothesis was further identified in clinical iCCA tissues. Numb^Low^ iCCA tissues had elevated NICD and Hes1 expression, particularly in nuclei. More frequent mitotic cells were observed in Numb^Low^/NICD^High^ tumors, indicating that low expression of Numb promotes the proliferation of iCCA cells by enhancing the Notch pathway (Fig. 7A).

## Discussion

The role of Numb in HPC expansion is still unclear. In the present study, we demonstrated that low Numb in HPCs promotes HPC expansion, liver cirrhosis, and iCCA development in response to chronic biliary injury. Mechanistically, Numb regulates HPC expansion and transformation by activating Notch signaling.

Numb, an important determinant of cell fate, is asymmetrically distributed in mitosis and produces different types of progeny cells. Asymmetric cell division is a basic process of stem cell development and differentiation [18]. Numb regulates changes in progenitors during lineage progression in sensory organ precursors and skeletal muscle progenitor cells in the embryo [31, 32], indicating that Numb may play an important role in development. To observe the function of Numb in liver development, we constructed liver-specific Numb deletion mice by the Alb-Cre/loxP system, in which Numb in the liver was constitutively ablated in the hepatoblast progeny cells. It has been reported that Numb is downregulated in HPCs during biliary regeneration, and Numb specifies HPC fate by regulating Notch and Wnt signaling [19]. The role of Numb in the liver involves regulating and balancing HPC differentiation and proliferation. Even though Numb plays a role in HPCs, HPCs are less than 1% and are almost static in the adult normal liver [13], and the absence of Numb in HPCs may not lead to the liver disease under normal conditions. However, almost all liver diseases are in a condition of chronic liver injury, and HPCs become activated under this condition. The role of Numb in the liver is more likely to regulate the proliferation and transformation of HPCs during chronic injury. Therefore, we performed 4 weeks of DDC treatment in Numb^−/−^ mice and found that Numb deletion promoted HPC expansion and liver fibrosis, indicating that Numb is a key protein that regulates HPC expansion.

iCCA cells share many stem cell markers with HPCs and have long been suggested to be derived from HPCs [16, 17]. We evaluated the role of Numb in iCCA development. Numb has been found to be downregulated in numerous malignancies, including HCC [33]. In our study, Numb expression was measured by IHC staining of a large cohort of 121 iCCA specimens, showing decreased Numb in human iCCA cells. To our surprise, our finding was contrary to the results of the TCGA database showing that Numb is upregulated in iCCA. One possible reason is that the number of patients in TCGA is small (less than 50). Another more important reason might be that in the adjacent noncancerous tissues used as a control, hepatocytes with positive Numb expression account for the majority compared to bile duct cells, which may lead to a bias of the actual expression of Numb in bile duct cells. Therefore, IHC may be a better way to detect Numb expression in bile duct cells. In addition, we found that Numb was downregulated in mouse iCCA and that liver-specific Numb deletion promoted iCCA development, suggesting that Numb plays a critical role as a tumor suppressor in iCCA. However, the mechanism of Numb decrease in iCCA remains unclear. A previous study has reported that Numb cooperates with the E3 ubiquitin ligase, MDM2, to promote the stability of p53 in cell lines [34, 35]. Numb may also be a ubiquitination target of MDM2 [34, 35]. Thus, the decrease in Numb in iCCA, as well as other malignancies, might be due to the overactivation of MDM2, which is frequently observed in various cancers.

Notch is an important oncogene that plays a crucial role in the development of the bile duct and iCCA. Interference with the Notch signaling pathway can lead to congenital biliary dysplasia, whereas overexpression of NICD can lead to iCCA [36, 37]. Correspondingly, our RNA-seq analysis demonstrated the activation of Notch signaling in iCCA in mice. The Numb protein is located upstream of the Notch and antagonizes the activity of the Notch pathway. Inactivation of Numb may cause abnormal cell differentiation and proliferation, leading to tumor formation. The exact mechanism by which Numb antagonizes the Notch signaling pathway is still unclear, but there are several possible mechanisms as follows [38–40]: (1) Numb affects the intracellular transport of Notch through endocytosis; (2) Numb promotes ubiquitination of the Notch receptor; and (3) Numb interacts directly with Notch. In our study, after observing intense high expression and nuclear accumulation of NICD in HuCCT1 and RBE cells after Numb silencing, we further confirmed that Numb can directly bind to the NICD, which might promote NICD degradation or prevent its nuclear translocation. In the canonical Notch signaling pathway, the Notch signaling pathway is initiated when transmembrane ligands bind to the extracellular domain of transmembrane Notch receptors [30]. Subsequently, NICD is released from the cell membrane mediated by γ-secretase. NICD translocates into the nucleus and binds to the CSL (CBF1, Su(H), and LAG1) transcription factor complex [30]. The nuclear translocation of NICD is an important process in Notch signaling and eventually leads to the activation of classical Notch target genes. In iCCA, Numb is likely involved in the regulation of nuclear translocation and degradation of NICD, thus regulating the Notch signaling pathway (as summarized in Fig. 7B).

In conclusion, we demonstrated the function of Numb in promoting HPC expansion, HPC malignant transformation, liver cirrhosis, and, ultimately, iCCA development in chronically injured livers. Mechanistically, Numb might inhibit Notch signaling by promoting the degradation or preventing the nuclear translocation of NICD.

## Supplementary information

Supplemental Material

## Data Availability

The datasets used and analyzed during the current study are available from the corresponding author on reasonable request.
