# Supplementary material for: Loss of Numb promotes hepatic progenitor expansion and intrahepatic cholangiocarcinoma by enhancing Notch signaling
Source: Cell Death Dis. 2021 Oct 19;12(11):966. doi: 10.1038/s41419-021-04263-w (PMC8526591; doi:10.1038/s41419-021-04263-w)
Supplement: Supplementary file 1 — Supplemental Material [file 41419_2021_4263_MOESM1_ESM.docx]

**Supplemental Material**

**Methods**

***Histology, immunohistochemistry and immunofluorescence staining***

Tissues harvested at defined times were fixed with 10% buffered formalin for 48 h and then processed for sectioning by embedding in paraffin. The paraffin sections (4 μm) were prepared for hematoxylin and eosin (H&E), Masson’s trichrome, sirius red, and immunohistochemistry or immunofluorescence staining. The antibodies used in this study are listed in Supplementary Table 1.

***Western blotting and C*oimmunoprecipitation**

Liver tissues or cultured cells were lysed in RIPA buffer and centrifuged at 13,000 rpm and 4 °C for 15 min. Western blotting was performed using standard protocols. The antibodies used in this study are listed in Supplementary Table 1.

For coimmunoprecipitation (co-IP) assays, anti-Numb or normal mouse/rabbit IgG were used as the primary antibody. Whole-cell lysates were incubated with primary antibodies at 4 °C followed by incubation with ProteinA/G Agarose.Agarose was collected and washed with lysis buffer. Equal volumes of each sample were analyzed by Western blot analysis. Kit for coimmunoprecipitation was purchased from Absin(Beijing, China) and coimmunoprecipitation was performed according to the manufacturer’s instructions.

***Cell culture***

The human iCCA cell lines HuCCT1 and RBE were both obtained from the American Type Culture Collection (ATCC) and were recently authenticated by STR profiling and tested for mycoplasma contamination. HuCCT1 and RBE were maintained in Roswell Park Memorial Institute 1640 medium (RPMI-1640 medium) (Gibco, NY, USA), supplemented with 10% fetal bovine serum, 2 mmol/L glutamine, and 100 U/mL penicillin/streptomycin. Cells were maintained at 37 °C in a 5% (volume per volume) CO_2_ atmosphere and subcultured every 3 days. For the immunofluorescence staining of cultured cells, cells were fixed with 10% buffered formalin and then subjected to standard fluorescence staining as described.

***Transfection and infection***

siNumb and corresponding negative control (NC) were purchased from Ribobio (Guangzhou, China) and cell transfection was performed according to the manufacturer’s instructions. The effects of the transient transfection were determined by western blotting analysis after 48 h. For stable inhibition of Numb expression, the sequence of shRNA was structured to lentiviral vector. shNumb and corresponding empty vectors were purchased from GeneChem (Shanghai, China). The screening drug puromycin was added 72 h after infection according to the manufacture’s instructions, and its concentration was maintained at 5 μg/mL for 24 h. The effects of the transfection were determined by western blotting analysis when the cells were grown to 90% conﬂuence. All the sequences described in this section are listed in Supplementary Table 2.

***Colony formation assay***

Cells (500 per well) were seeded into six-well plates and cultured in a humidiﬁed incubator at 37 °C for 15 days, with medium changes every 3 days. Then, the colonies were ﬁxed with 4% paraformaldehyde for 10 min, stained with 0.1% crystal violet for 30 min, and the colonies were counted. All experiments were performed at least three times independently.

***Transwell assay***

Transwell migration chambers (24-well) (Corning, NY, USA) was pre-coated with a thin layer of Matrigel Basement Membrane Matrix (BD Biosciences, Shanghai, China). Suspensions of 8 × 10^4^ cells in 200 μL of serum free medium were added to the upper chamber, and medium containing 10% FBS was added to the lower chamber to serve as the chemoattractant. The chambers were incubated at 37 °C in 5% CO_2_ for 24 h, and the cells in the upper chamber were then removed with cotton swabs. The migrated cells attached to the lower chamber were ﬁxed with 4% paraformaldehyde, stained with 0.1% crystal violet and quantiﬁed by light microscopy. Cells from ﬁve random ﬁelds were counted.

***Sphere formation***

Cells were plated at a density of 5 × 10^3^ cells per well in 6-well Ultra-Low Attachment plates (Corning). After 15 days of growth in selective serum-free advanced Dulbecco’s modified Eagle’s medium (DMEM) / F12 (Thermo Fisher Scientific, Waltham, MA, USA) supplemented with 2% B-27 supplement without vitamin A (Life Technologies, Carlsbad, CA, USA), recombinant human bFGF (20 ng/mL), bEGF (20 ng/mL) and heparin (4 μg/mL) were added.

***Wound-healing assay***

The cells were seeded in 6-well plates (Corning) and grown to 90% conﬂuence in complete medium. The artiﬁcial wound was prepared by scraping the conﬂuent cell monolayer with a 200-µL pipette tip and then washing with phosphate-buffered saline (PBS) to remove the isolated cells. The cells were grown in serum-free medium at 37 °C with 5% CO_2_ for 24 h. Cell migration was assessed by microscopy and analyzed objectively with ImageJ 1.5.1. The wound closure percentages were calculated using the following formula: 1−[24-h area / 0−h area]. Three independent assays were photographed and quantiﬁed.

**
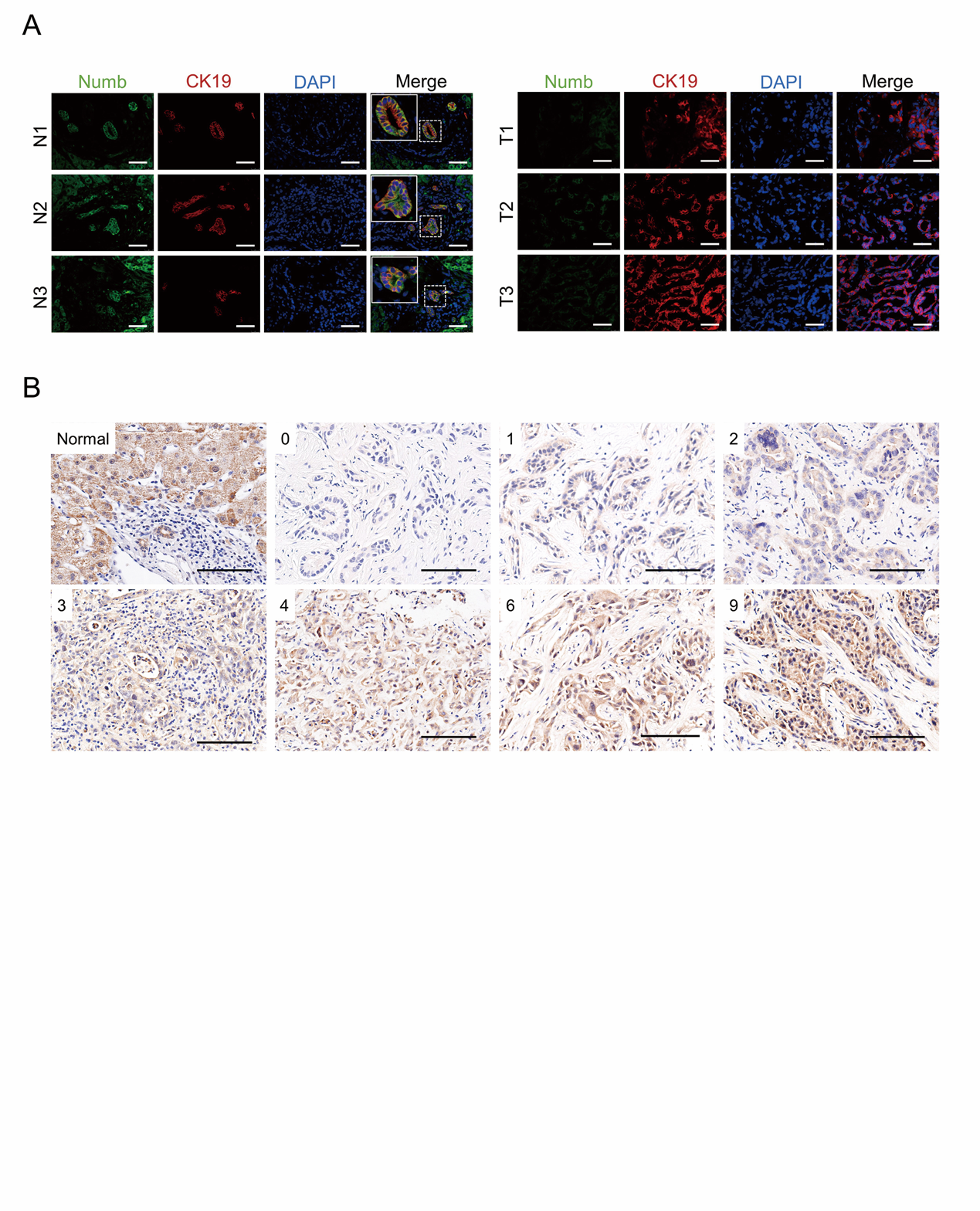
**

**Supplementary Figure. S1.**

(A) Immunofluorescence staining of Numb and CK19 in iCCA specimens and paired normal tissues. Scale bar, 100 μm. (B) According to the percentage of Numb+ tumor cells in specific areas, specimens were assigned to the following categories: 0, <5%; 1, 5%-25%; 2, 25%-70%; 3, >75%. The intensity of IHC staining was categorized as follows: 1, weak; 2, moderate; and 3, intense. The final score of each specimen was the product of the percentage of Numb+ tumor cells multiplied by the intensity of IHC staining. Therefore, the potential final scores were 0, 1, 2, 3, 4, 6, and 9, and the corresponding figures are presented. An IHC score of 0, 1, 2, or 3 was considered low expression, and a score of 4, 6, or 9 was considered high expression. Scale bar, 100 μm.


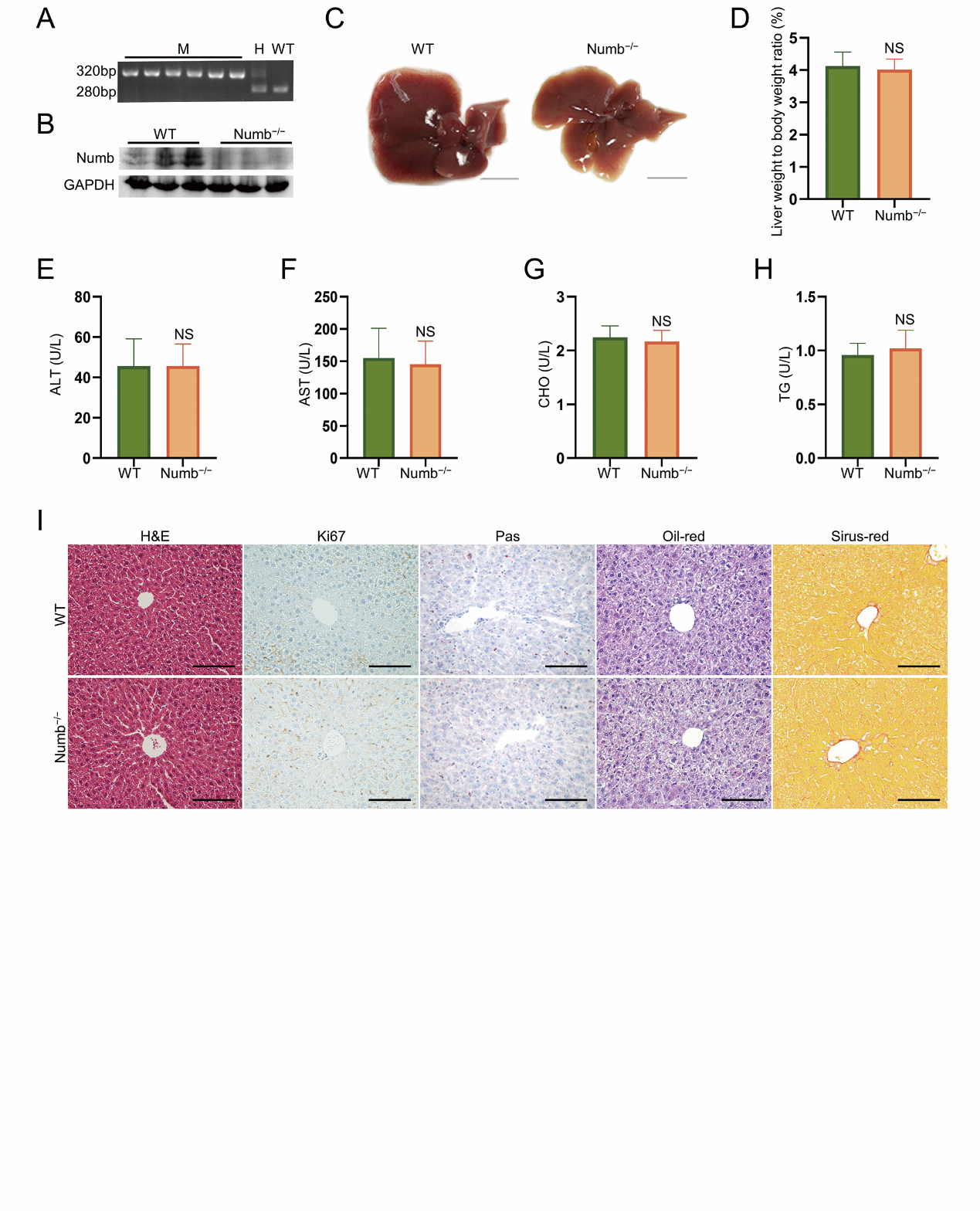


**Supplementary Figure. S2.**

(A) Genotyping test (M, mutant; H, Heterozygote). (B) Western blotting analysis verified the knockout efficiency. (C, D) Gross views and the liver weight/body weight ratios of WT and Numb^-/-^ livers. (E-H) Biochemical indexes of ALT, AST, CHO and TG. (I) H&E, Ki67, PAS, Oil-red O, and Sirus-red staining, respectively (40X). All data represent the mean ± SD; N=6.


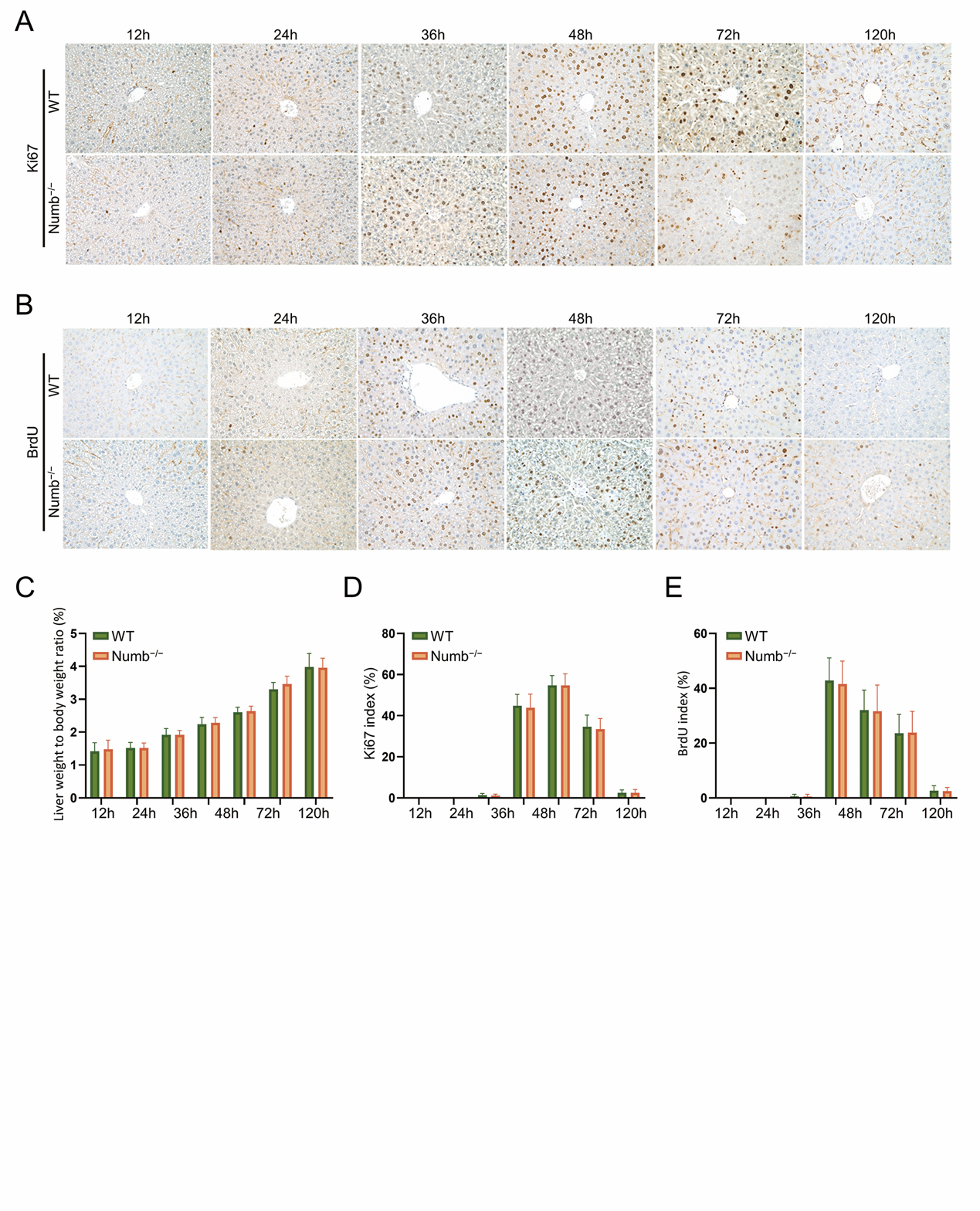


**Supplementary Figure. S3.**

(A, B) Immunohistochemistry staining of Ki67 and BrdU shows that the proliferation rates in WT mice and Numb^-/-^mice after PH (40X). (C-E) The liver weight/body weight ratios, Ki67 and BrdU index in WT and Numb^-/-^ livers after PH. All data represent the mean ± SD; n=6.


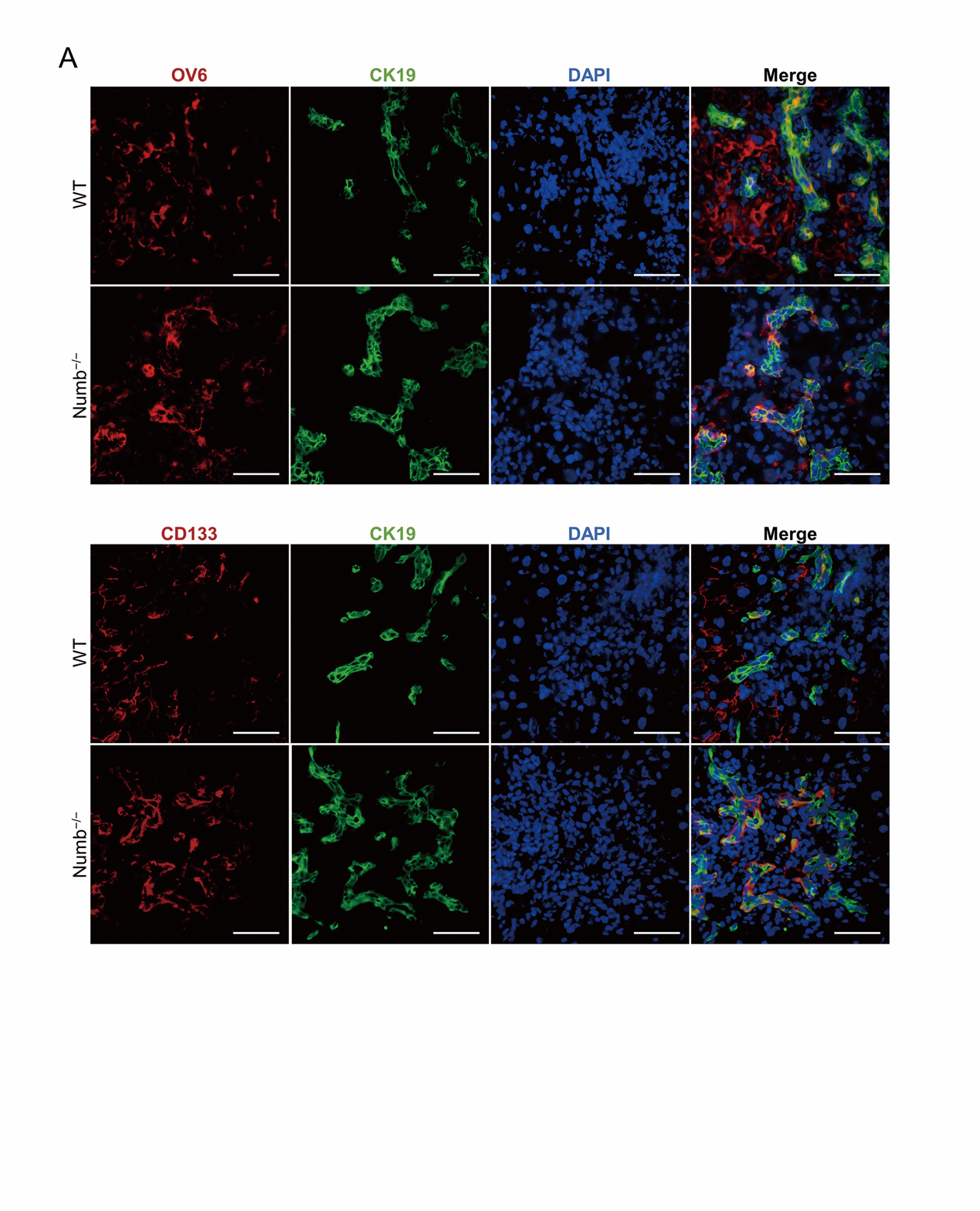


**Supplementary Figure. S4.**

(A) Immunofluorescence staining exhibits that the expansing CK19-positive cells were HPCs marked by OV6 and CD133. Scale bar, 50 μm.


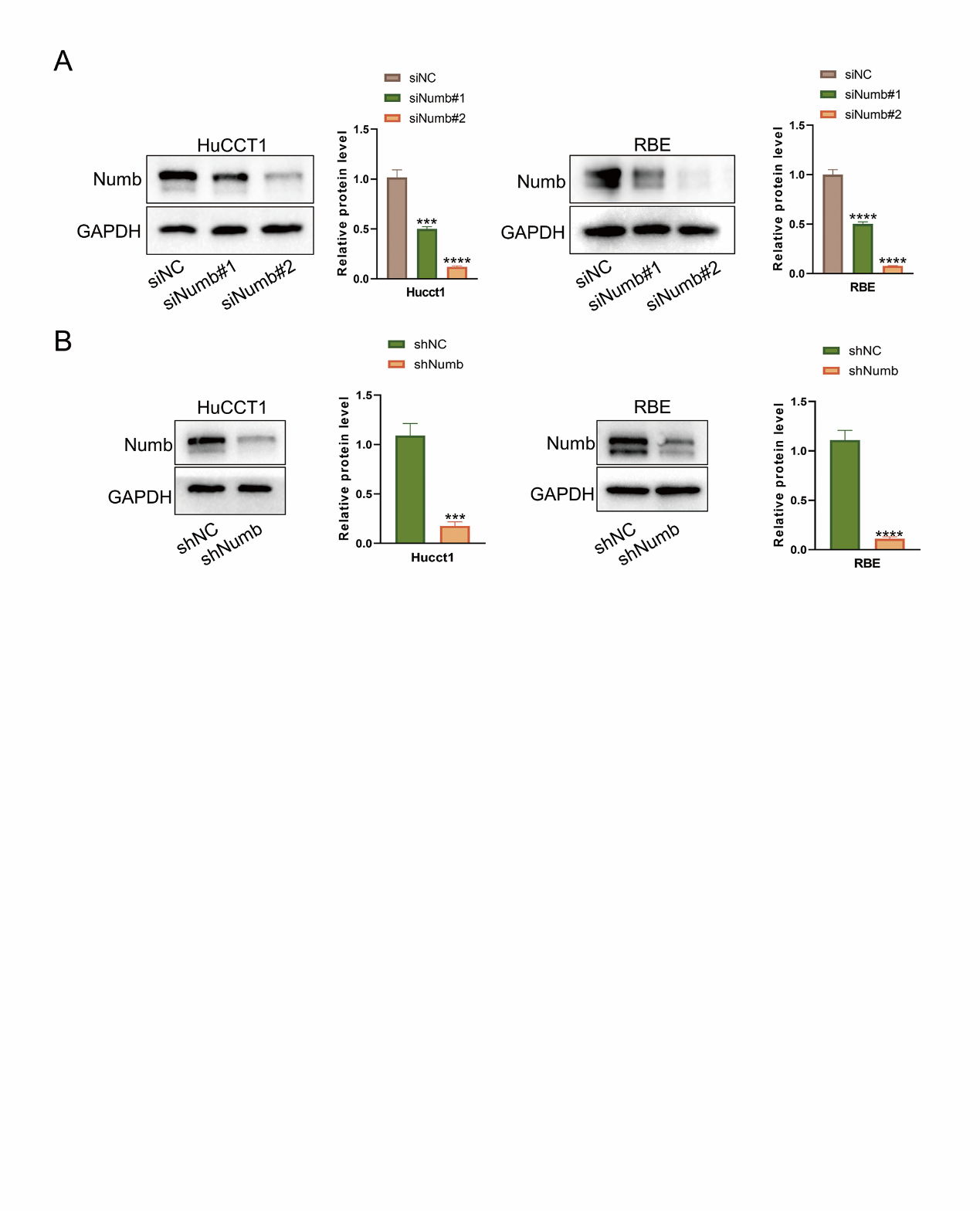


**Supplementary Figure. S5.**

(A) Western blotting was performed to determine Numb expression after transfection with siNC and siNumb in Hucct1 and RBE cells. (B) Western blotting was performed to determine Numb expression after transfection with shNC and shNumb structured to lentiviral vector in Hucct1 and RBE cells. The corresponding statistical results beside the image. All data represent the mean ± SD of at least three independent experiments; ****P* < 0.001.


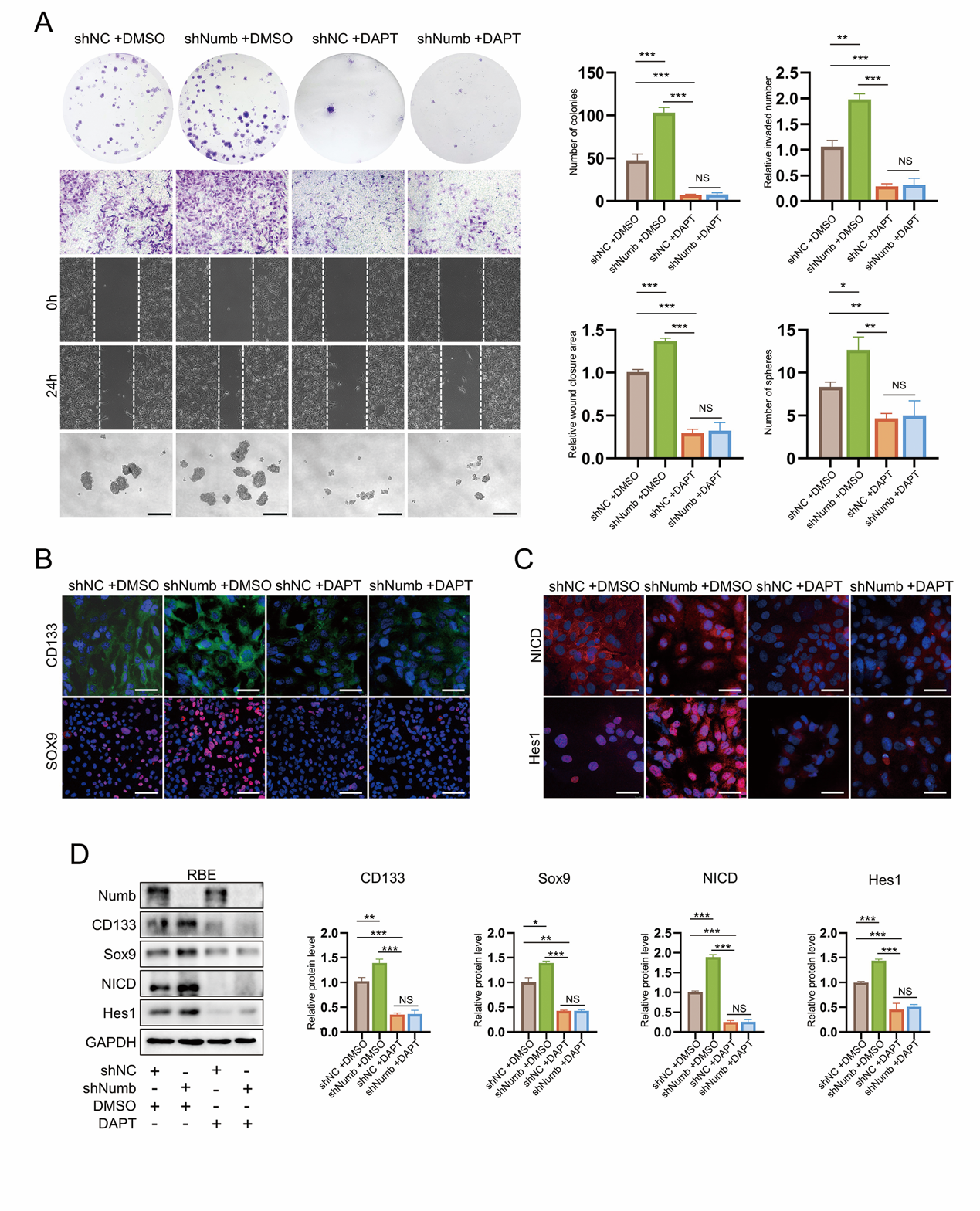


**Supplementary Figure. S6.**

(A) The cell proliferation, metastasis and stemness of RBE cells were measured by colony formation, migration, wound healing and sphere formation assays after DAPT treatment at a dose of 150 μM (the control group was treated with the same volume of DMSO without DAPT). Scale bar, 200 μm. (B) Immonofluorescence staining of HPC markers (CD133 and SOX9) in RBE cells. Scale bar, 50 μm (CD133) and 100 μm (SOX9), respectively. (C) Immonofluorescence staining of the key proteins of Notch signaling, NICD and Hes1 in RBE cells. Scale bar, 50 μm. (D) Western blotting displaying that the up-regulated HPC markers and the downstream factors of Notch after Numb silencing were reversed after DAPT treatment in RBE cells.

**Supplementary Table 1. Antibodies**

| **Antibody** | **Company** | **Identifier (Catalog No.)** |
| --- | --- | --- |
| Numb | Cell Signaling Technology | 2756 |
| CD133 | Proteintech Group | 18470-1-AP |
| CK19 | Abcam | 133496 |
| CK19 | Huabio | EM1901-75 |
| OV6 | Novus | 18961 |
| Hes1 | Huabio | ET1610-97 |
| Hey1 | Thermo Fisher Scientific | PA5-23484 |
| CD24 | Huabio | 0804-3 |
| CD44 | R&D Systems | AF6127 |
| NICD | Cell Signaling Technology | 4147 |
| Notch1 | Cell Signaling Technology | 3608 |
| Notch2 | Cell Signaling Technology | 5732 |
| Notch3 | Cell Signaling Technology | 5267 |
| Sox9 | Huabio | ET1611-56 |
| Ki67 | Thermo Fisher Scientific | RM-9106-S1 |
| HNF4α | Huabio | ET1611-43 |
| CDK1 | Abcam | Ab71939 |
| CDK2 | Cell Signaling Technology | 2546 |
| GAPDH | Huabio | R1210-1 |

**Supplementary Table 2**

**Sequences for Gene Silencing**

| **Gene Name** | **Forward** |
| --- | --- |
| siNumb-1 | GAAGGATCATTCCGTGTCA |
| siNumb-2 | GACTCAGAGTTGTGGATGA |
| shNumb | gcCATGTAGAAGTTGATGAAT |

**Sequences for Genotyping analysis**

| **Gene Name** | **Forward** | **Reverse** |
| --- | --- | --- |
| Numb | GAAGGAGCCTTCCAAAATCGTATTC | AGGCTTCTGGGAAACCTCACTTACTC |
| Cre | CACCCTGTTACGTATAGCCGCCTAGGCACCAGGGTCTCAT | GAGTCATCCTTAGCGCCGTATCACGGTTGGCCTTAGGGTT |

**Supplementary Table 3. Correlations between Numb expression and clinicopathological features of ICC patients**

|  | Brg1 expression | | |  |
| --- | --- | --- | --- | --- |
| Variables | Low (n=73) |  | High (n=44) | *P value* |
| Age (yr) | 56.36±10.28 |  | 56.93±10.23 | 0.716 |
| Gender (M/F) | 33/40 |  | 28/16 | 0.054 |
| T-bil (umol/dL) | 13.73±7.31 |  | 13.51±6.57 | 0.872 |
| ALB (g/L) | 41.66±5.74 |  | 43.23±3.78 | 0.108 |
| ALT (IU/L) | 58.82±12.53 |  | 42.73±8.44 | 0.452 |
| AST | 58.47±11.26 |  | 36.57±35.64 | 0.222 |
| WBC | 7.91±5.42 |  | 6.82±2.45 | 0.213 |
| HGB | 131.12±17.14 |  | 128.61±16.87 | 0.984 |
| CREA | 69.68±12.84 |  | 69.63±12.34 | 0.442 |
| Tumor size (cm) | 6.20±2.75 |  | 6.41±2.43 | 0.671 |
| Tumor number  (single/multiple) | 65/8 |  | 36/8 | 0.682 |
| Tumor differentiation  (well-mod/poorly) | 24/49 |  | 13/31 | **0.035** |
| Vascular invasion  (yes/no) | 9/64 |  | 8/36 | 0.710 |
| Mean time to recurrence  (months) | 11.00±10.24 |  | 20.69±18.76 | **＜0.001** |
